# Taurine as a Protective Metabolite in Radiation-Induced Liver Disease: Evidence from ^1^H NMR Metabolomics

**DOI:** 10.1021/acs.jproteome.5c00398

**Published:** 2025-08-11

**Authors:** Yi-Hsiu Chung, Chi-Chang Weng, Fujie Jhang, Gigin Lin, Ching-Fang Yu, Fang-Hsin Chen

**Affiliations:** † Department of Medical Research and Development, Research Division, 38014 Chang Gung Memorial Hospital at Linkou, Taoyuan 33382, Taiwan; ‡ Department of Medical Imaging and Radiological Sciences, 56081 Chang Gung University, Taoyuan 33302, Taiwan; § Department of Medical Imaging and Intervention, 38014 Chang Gung Memorial Hospital at Linkou, Taoyuan 33382, Taiwan; ∥ Clinical Metabolomics Core and Imaging Core Laboratory, Institute for Radiological Research, Chang Gung Memorial Hospital at Linkou, 56081 Chang Gung University, Taoyuan 33382, Taiwan; ⊥ Research Center for Radiation Medicine, 56081 Chang Gung University, Taoyuan 33302, Taiwan; # Department of Radiation Oncology, Chang Gung Memorial Hospital Linkou Branch, Taoyuan 33382, Taiwan; ∇ Institute of Nuclear Engineering and Science, 34881 National Tsing Hua University, Hsinchu 300044, Taiwan

**Keywords:** ^1^H NMR metabolomics, radiation-induced liver diseases, taurine, preclinical study

## Abstract

Radiation therapy for liver and upper abdominal malignancies is associated with a high risk of radiation-induced liver disease (RILD), often involving metabolic disturbances. This study aimed to characterize metabolomic alterations in a murine RILD model using ^1^H-nuclear magnetic resonance (NMR) spectroscopy. RILD was induced in mice via 15 Gy hepatic irradiation. Liver tissues were subjected to time-course metabolomic profiling using ^1^H NMR. Validation was performed through liver enzyme assays, histological analysis, and qPCR. Taurine was selected for therapeutic evaluation based on metabolomic findings. Significant reductions in glutathione and pyruvate levels were observed in the acute stage, whereas increases in phenylalanine and tyrosine levels were observed in the chronic stage. These metabolite alterations were correlated with reactive oxygen species (ROS) production, glycolysis modulation, and fibrosis progression in RILD. Notably, taurine levels increased during the acute stage but decreased during the chronic stage. Furthermore, in mice at 12 weeks postirradiation, taurine supplementation maintained liver enzyme activity, cytokine profiles, and taurine metabolism at levels similar to those of the control group. In conclusion, this study revealed dynamic metabolic changes associated with physiological alterations in a murine RILD model and identified, through ^1^H NMR metabolomics, taurine as a potential target for alleviating nonclassical RILD symptoms.

## Graphical abstract

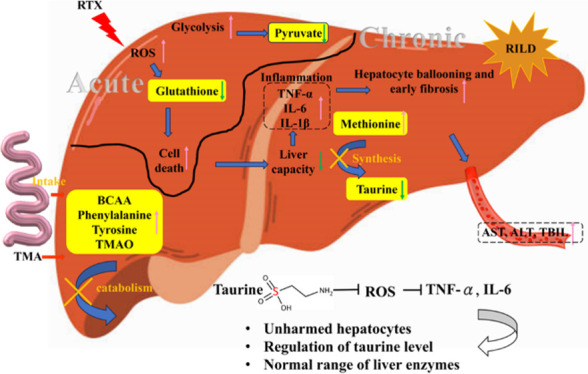

## Introduction

Radiation-induced liver disease (RILD) is a major dose-limiting complication associated with radiation therapy (RT) for liver and upper abdominal malignancies,^1^ particularly in patients with unresectable tumors due to their size or anatomical location.^2^ Modern radiotherapy techniques, such as stereotactic body radiation therapy (SBRT),^3^ have improved the precision of tumor targeting and expanded treatment options for patients who are not surgical candidates. However, despite these advancements, the risk of RILD remains a significant concern, as it can occur in over 30% of patients receiving SBRT.^4^ This potential for liver toxicity imposes strict constraints on the maximum deliverable radiation dose, ultimately limiting the therapeutic efficacy of RT in clinical settings.

RILD can be divided into classical and nonclassical forms.^5^ Classical RILD typically manifests 2–12 weeks after radiation therapy, particularly when the whole-liver tolerance dose (30–35 Gy) is exceeded, whereas nonclassical RILD can occur within 1–12 weeks. Classical RILD is characterized by a 2-fold elevation in alkaline phosphatase levels above the upper normal limit and severe liver toxicity, with symptoms including fatigue, abdominal pain, anicteric hepatomegaly, and ascites. In the chronic stage, typically within 3–5 months, patients may develop liver fibrosis and failure.^6^ Conversely, nonclassical RILD is defined by liver transaminase levels exceeding five times the upper normal limit, a Child-Pugh score increase of ≥2, or the absence of classical RILD symptoms.

Beyond biochemical enzyme levels, recent studies have identified additional metabolites as potential indicators of liver damage.^7–9^ For example, succinate is proposed to regulate hepatic stellate cells in liver fibrosis.^10^ In our previous study, we demonstrated that elevated levels of pyruvate and glutamate in the liver parenchyma 72 h postirradiation are correlated with alterations in glycolysis and the tricarboxylic acid (TCA) cycle.^11^ The metabolites released from the damaged liver could have great potential for diagnosing hepatic injury. The mechanism of liver fibrosis induced by various toxicities, including radiation, has been studied.^12,13^ Hepatic stellate cell (HSC) activation is mediated by cytokines released from apoptotic hepatocytes, and activated HSCs are characterized by the high expression of α-smooth muscle actin, abundant deposition of extracellular matrix, and loss of lipid droplets, which induces liver fibrosis.^14^ However, the dynamic metabolic changes from the acute stage to the chronic stage of radiation-induced liver damage remain unknown. Elucidating the dynamic alterations in metabolite profiles from the acute to chronic stages of RILD is pivotal not only for prognosticating the likelihood of irreversible hepatic injury—such as fibrosis and liver failure—but also for identifying critical therapeutic windows during which targeted modulation of metabolic pathways may mitigate or even prevent pathological progression.

Metabolomics is a current research trend because the impact of altered metabolites may reflect upstream proteomic, transcriptomic, and even genomic changes.^15^ In this study, we utilized ^1^H-nuclear magnetic resonance (NMR) spectroscopy, a powerful and reliable tool in metabolomics, to analyze multiple metabolites and molecules and examine the correlation of metabolite changes with physiological evidence. The aim of this study was to explore the dynamic changes in metabolites in RILD via a nontargeted ^1^H NMR system to elucidate the underlying mechanism of RILD. The findings indicated that key metabolites may serve as potential targets for preventing or alleviating radiation-induced adverse effects in liver tissue.

## Materials and Methods

### Animal Experimental Design

To investigate the temporal progression of RILD, normal eight-week-old C57BL/6 mice (National Laboratory Animal Center, Taiwan) were exposed to 15 Gy of irradiation on the upper right lobe of the liver. Mice were sacrificed 3 h, 24 h, 8 weeks, and 12 weeks postirradiation. In this study, we defined the acute phase as the early time points (3 and 24 h postirradiation), representing the oxidative response and glycolysis. The chronic phase was defined as the later time points (8 and 12 weeks postirradiation), reflecting long-term tissue inflammatory and fibrotic progression.^13,16,17^ To assess liver function, blood was collected either by cardiac puncture before sacrifice or through the submandibular method for subsequent analysis. Plasma was then isolated by centrifugation. The right lobe of the liver was excised and harvested for ^1^H NMR spectroscopy, qPCR, and histological analysis.

Two groups of mice were given taurine supplements through drinking water, with one group receiving supplementation prior to radiation therapy (pretaurine group) and the other receiving supplementation after irradiation (post-taurine group), to examine the impact of taurine on RILD development. All experimental animal protocols were performed in compliance with the animal guidelines approved by the Institutional Animal Care and Use Committee of Chang Gung Memorial Hospital, Taiwan (IACUC 2016010701 and IACUC 2023121819). A flowchart is provided in Figure 1.

**Figure 1:**
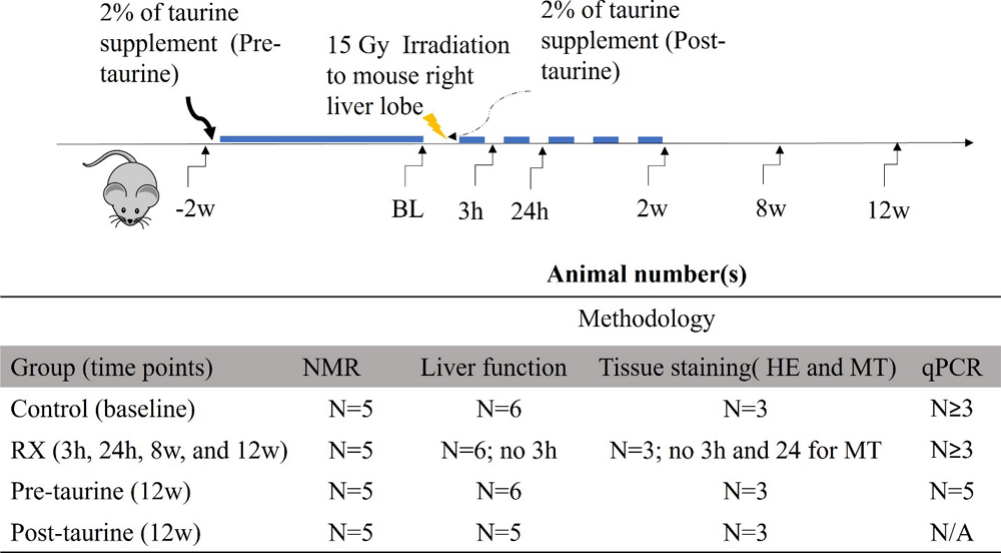
Experimental design involved grouping animals on the basis of postirradiation time points of 3 h, 24 h, 8 weeks, and 12 weeks. Mice received a 2% taurine supplement through drinking water for 2 weeks before and after irradiation (pretaurine treatment group (blue line) and post-taurine treatment group (dotted line), respectively). The numbers of animals included in the NMR study, liver function analysis, histological staining, and cytokine analysis are listed. The animals in the control group were not irradiated.

### Radiation Protocol

Mice under anesthesia with isoflurane (2%) were restrained using adhesive tape before irradiation. To consider the impact of anesthetic drugs on metabolism, mice were awakened but restrained on the stage during irradiation.^18^ The irradiation protocol was followed as described in our previous report with minor modifications.^19^ The irradiation field (10 × 10 mm) targeted the upper right region of the xiphoid process in mice positioned supine, encompassing part of the liver parenchyma (Supplemental Data 1), and the mice were exposed to 15 Gy of 6-MV X-ray beams from a linear accelerator at a 6 Gy/min dose rate, with a 0.5 cm bolus covering the skin surface.

### Tissue Extraction and ^1^H NMR Spectroscopy

The mice were given ad libitum access to food and water before being sacrificed at specific times. The restrictedly irradiated liver tissues were collected and stored at–80 °C to preserve sample integrity for subsequent analysis. A total of 40 mg of liver tissue was homogenized with 1.6 mL of a mixture of methanol and water (methanol: ddH_2_O = 5:1). After the homogenates were transferred to a glass culture tube, 625 μL of chloroform was added, followed by vortexing for 30 s. An additional 625 μL of a mixture of chloroform and ddH2O (1:1) was added, after which the mixture was placed on ice for 30 min. Then, the samples were centrifuged at 12,000 rpm at 4 °C for 30 min. After centrifugation, the upper aqueous layer was retrieved and dried under a stream of nitrogen. The aqueous extracts were reconstituted with 200 μL of CDCl_3_/0.025% tetramethyl silane or D_2_O/0.005% (trimethylsilyl)­propanoic acid (TSP). The reconstituted aqueous extracts were then centrifuged at 12,000 rpm for 5 min at 4 °C, and the supernatants were retained. An aliquot (180 μL) of the reconstituted lipid or aqueous extract was slowly transferred, to avoid air bubbles, into a 3 mm NMR tube for subsequent NMR analysis. ^1^H NMR spectra for aqueous liver extracts were acquired using a Bruker Avance III HD 600 MHz spectrometer (Bruker Biospin GmbH, Karlsruhe, Germany) operating with a 14.1 T magnet and equipped with a 5 mm inverse triple resonance probe. Acquisition was carried out using a NOESY pulse sequence, which is widely used for tissue NMR analysis in the Bruker system. The temperature of the spectrometer was maintained at 283 K. The standard setting for metabolites in aqueous tissue extracts was applied, with the following parameters: acquisition time, 2.7 s; spectral window, 20 ppm; spin–echo delay, 300 ms; and number of scans, 128 with 64k data points.

### Metabolite Analysis

The metabolite analysis was performed as described in a previous study.^11^ Briefly, a standard internal reference, TSP, was used to calibrate metabolic concentrations in the aqueous phase. Each metabolite was identified and quantified using Chenomx NMR Suite software (Chenomx, Inc., Edmonton, AB, Canada). The ratio of each metabolite in the sample was calculated by dividing the amount of a metabolite by the amount of metabolites in the whole spectrum. Metabolite data expressed as ratios were statistically analyzed using MetaboAnalyst 6.0 web software. A statistical analysis (one factor) module was used to compare data after normalization by the sum, log transformation, and Pareto scaling for the data set approximate to a normal distribution. A significant difference was considered under three conditions: a fold change greater than 1.2 or less than 0.8, a p value in the Wilcoxon rank-sum test less than 0.05 for a small number of samples, and a variable importance in projection (VIP) score >1.1 in the partial least-squares discriminant analysis (PLS-DA) model. A pathway analysis module was used to examine the metabolic pathways associated with significant metabolites. Pathways were deemed significantly important if their p value was less than 0.05 and their impact value was greater than 0.2.

### Liver Function Test

Aliquots (150 μL) of blood samples were collected from the cardiac or submandibular veins of mice at various time points (preirradiation and postirradiation day 1, week 8, and week 12) and placed into heparin blood collection microtubes for biochemical enzyme analysis. The plasma was isolated via centrifugation at 3000 rcf for 20 min. The levels of liver enzymes, including aspartate aminotransferase (ALT) and alanine aminotransferase (AST), and total bilirubin (TBIL) were measured using veterinary chemistry reagent discs designed specifically for animals (cat 001-3GYC, AmiShield, Taiwan).

### Histopathological Analysis of Liver Damage

Liver tissues were obtained from mice that received radiation at 3 and 24 h, as well as, at 8 and 12 weeks postirradiation. Tissues from unirradiated mice served as the control group. The tissues were stored in 4% paraformaldehyde, embedded in paraffin, and sectioned (3 μm thick) for routine hematoxylin–eosin (H&E) and Masson’s trichrome (MT) staining. The liver tissues were analyzed for radiation damage. The H&E- and Masson’s trichrome-stained sections were imaged with a TissueFAXS PLUS system (TissueGnostics Inc., Vienna, Austria). The fraction of collagen visualized by Masson’s trichrome staining was calculated using ImageJ 1.5v software. The results are presented as bar graphs showing the percentage of the Masson-positive area to the total area.^20^

### Assessment of Inflammation and Fibrotic Cytokines by Real-Time PCR

Total RNA was isolated using Trizol reagent (Invitrogen) and then reverse transcribed into cDNA using an Omniscript reverse transcriptase kit (Qiagen, Hilden, Germany) in accordance with the manufacturer’s protocol. The gene expression level in liver lobes was analyzed using a LightCycler 480 SYBR Green I Master Mix (Roche, Basel, Switzerland) and a CFX Connect real-time PCR system (Bio-Rad, Hercules, CA). The fold change in gene expression in each group was calculated as the difference (ΔΔCt) compared with the nonirradiated control group, where Ct is the threshold value. The sequences of the primers used were as follows: β-actin forward primer, ACCCTAAGGCCAACCGTGAA; β-actin reverse primer, ATGGCGTGAGGGAGAGCATAG; TNF-α forward primer, CACGTCGTAGCA AACCACCAAGTCGA; TNF-α reverse primer, TGGGAGTAGACAAGGTAC AACCC; IL-6 forward primer, AGTTGCCTTCTTGGGACTGA; IL-6 reverse primer, TCCACGATTTCCCAGAGAAC; IL-1β forward primer, TGAAGCAGCTATGGCAACTG; IL-1β reverse primer, TTGTTGATGTGCTGCTGTGA; IL-18 forward primer, CAGGCCTGACATCTTCTGCAA; and IL-18 reverse primer, TCTGACATGGCAGCCATTGT.

### Taurine Supplementation Study

To study the effects of taurine on the prevention and treatment of radiation-induced liver damage, 12 eight-week-old C57BL/6 mice were divided into two groups: pretaurine treatment and post-taurine treatment. The pretaurine treatment group was given a 2% taurine supplement (Signa-Aldrich T8691, Merck Life Science Pty Ltd., Germany) in their drinking water for 2 weeks before irradiation, and the post-taurine treatment group was given a 2% taurine supplement for 2 weeks immediately after irradiation. The efficacy of the taurine supplements was assessed with the above-mentioned measurements 12 weeks postirradiation and compared with that of the control (baseline).

### Statistical Analysis

^1^H NMR metabolomics data (postirradiation data and baseline data) were compared using the nonparametric Mann–Whitney U test. For the qPCR data, changes in the expression of each of the investigated genes were determined by calculating the ΔΔCt values and then compared using unpaired one-way ANOVA. All analyses were performed using GraphPad Prism 8.01 (GraphPad Inc., San Diego, CA, USA). A *p*-value of less than 0.05 was considered statistically significant.

## Results

### Alterations in Metabolites in the Acute and Chronic Stages

To evaluate the dynamic changes in metabolites in irradiated livers, metabolites from the control group and experimental groups at various time points were analyzed using PCA and heatmaps through ^1^H NMR spectra. To obtain a more reliable statistical analysis and specific loadings, a consolidated PLS-DA model was used to discriminate between samples from the control group and all experimental time points. The plot for principal component 1 versus principal component 2 clearly showed separations between the control group and all experimental time points, as shown in Figure 2A. Three distinct divisions of metabolites are identified: the control group, the acute phase, and the chronic phase of RILD, providing a more precise representation of the progression of RILD. Heatmaps comparing metabolite expression patterns in liver tissues between the experimental groups at various time points and the control group are shown in Figure 2B. The metabolomes of the liver tissues significantly differed between the control and irradiated mice at each time point. Compared with those in liver tissues from control mice, the metabolites in liver tissues from mice that received 15 Gy irradiation were significantly altered at 3 h, 24 h, 8 weeks, and 12 weeks. The significant changes in metabolites after liver irradiation are listed in Table 1. The levels of taurine significantly changed dynamically in all the groups. The taurine levels increased in the acute stage, with FC = 2.46 at 3 h and FC = 2.13 at twenty-4 h postirradiation, and decreased in the late stage, with FC = 0.28 and 0.28 at eight and 12 weeks postirradiation, respectively. These findings suggest that taurine may be involved in the dynamic physiological response to RILD, potentially reflecting either a compensatory mechanism in the acute phase^21^ or compromised liver function in the later stages. Additionally, distinct changes in other metabolites were also observed in the acute and chronic stages of RILD progression. Decreased glutathione levels, FC = 0.22 in the liver, were found after irradiation for twenty-4 h, an effect that may be related to ROS production.^22^ The pyruvate levels decreased in the acute stage (FC = 0.16 for 3 h and 0.17 for 24 h), indicating alterations in glycolysis and the TCA cycle. In contrast, increased levels of O-phosphocholine, betaine, and methionine in the acute stage of RILD could be associated with impeded methionine metabolism resulting from hepatocyte damage.^23^ In the chronic stage for 12 weeks, the levels of metabolites such as valine, isoleucine, and leucine (BCAAs), trimethylamine N-oxide (TMAO), phenylalanine, and tyrosine in liver tissues significantly increased, effects that were correlated with liver injury and fibrosis.

**Table 1:** Changes in Metabolites at Various Time Points Compared with Baseline (Control)^a^

metabolite	fold change (RT3h/ctrl)	VIP > 1.1	Wilcox-rank test (*p*-value)	FDR (*q*-value)
pyruvate	0.16131	2.1369	0.007937**	0.038095*
methionine	4.0525	1.7128	0.015873*	0.063492
betaine	2.6005	1.4962	0.007937**	0.038095*
creatine	3.2429	1.4786	0.055556	0.12121
O-phosphocholine	2.902	1.3167	0.031746*	0.095238
taurine	2.4613	1.3036	0.031746*	0.095238
acetate	2.2267	1.2204	0.055556	0.12121
alanine	1.8762	1.2105	0.007937**	0.038095*
creatinine	2.3988	1.1006	0.095238	0.17582
	fold change (RT24h/ctrl)			
pyruvate	0.17469	1.8628	0.007937**	0.027211*
methionine	5.0114	1.821	0.007937**	0.027211*
glutathione	0.21973	1.7905	0.007937**	0.027211*
creatine	2.7046	1.3572	0.007937**	0.027211*
betaine	2.0141	1.1327	0.007937**	0.027211*
creatinine	2.0589	1.1135	0.031746*	0.058608
O-phosphocholine	2.3715	1.1012	0.031746*	0.058608
taurine	2.1264	1.100	0.015873*	0.042328*
	fold change (RT8w/ctrl)			
taurine	0.28492	1.6869	0.004329**	0.01039*
valine	2.4646	1.4923	0.004329**	0.01039*
trimethylamine N-oxide	2.3933	1.4687	0.004329**	0.01039*
fumarate	2.428	1.4675	0.004329**	0.01039*
mannose	2.4474	1.4204	0.004329**	0.01039*
isoleucine	2.1498	1.3615	0.004329**	0.01039*
leucine	2.1279	1.3273	0.004329**	0.01039*
tyrosine	1.976	1.2872	0.004329**	0.01039*
phenylalanine	1.8904	1.2278	0.004329**	0.01039*
	fold change (RT12w/ctrl)			
taurine	0.28144	1.6687	0.007937**	0.019048*
trimethylamine N-oxide	2.8849	1.575	0.007937**	0.019048*
valine	2.419	1.4442	0.007937**	0.019048*
methionine	2.4887	1.3975	0.015873*	0.034632*
isoleucine	2.2065	1.3725	0.007937**	0.019048*
leucine	2.0435	1.285	0.007937**	0.019048*
phenylalanine	2.0351	1.2696	0.007937**	0.019048*
tyrosine	1.9324	1.2107	0.007937**	0.019048*

a *, p and *q* < 0.05. **, *p* and *q* < 0.01.

**Figure 2:**
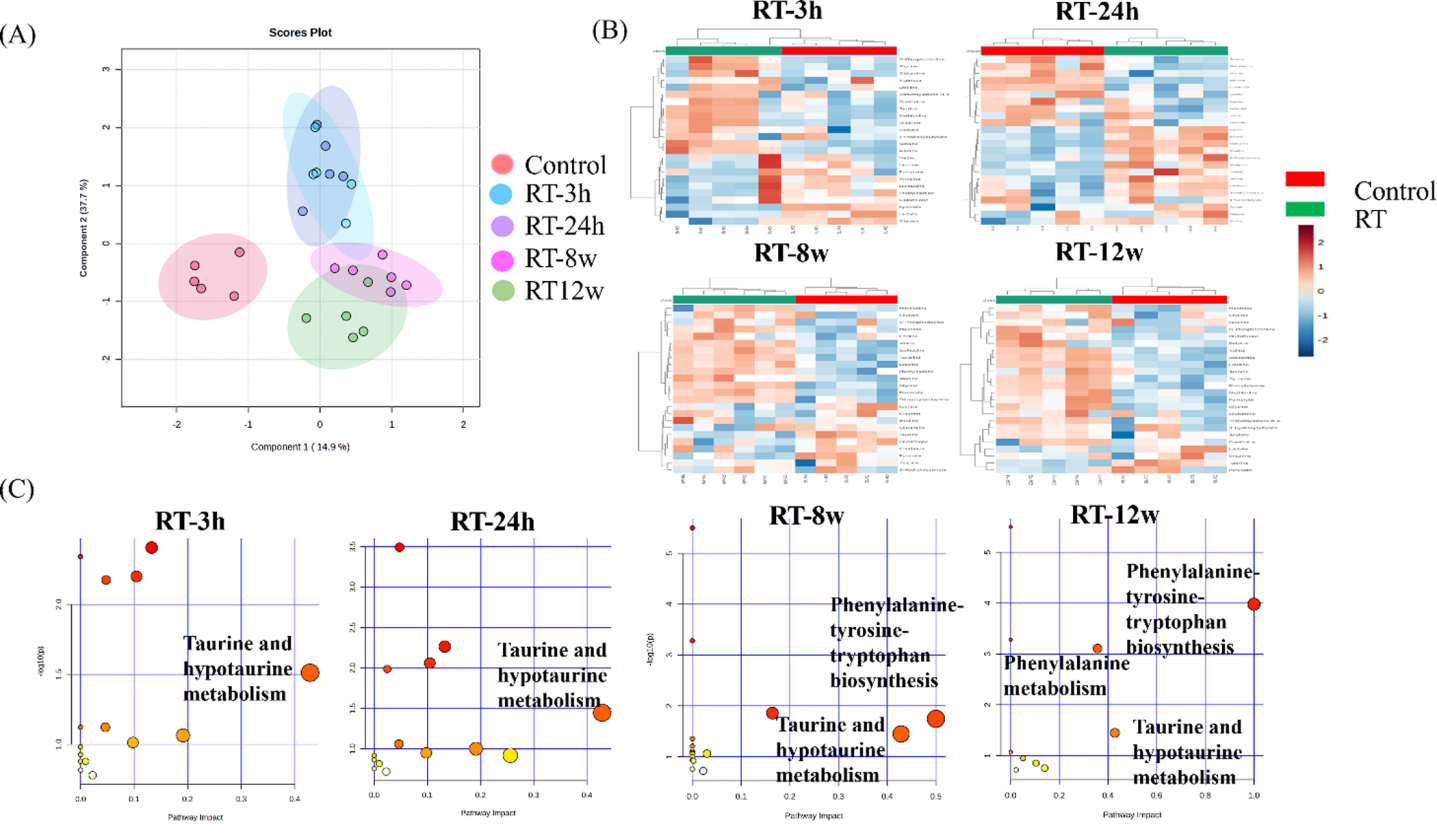
Metabolite analysis using an NMR metabolomics platform. (A) A consolidated PLS-DA plot encompassing both the control group and all experimental time points was displayed. Three distinct divisions of metabolites are identified: the control group, the acute phase, and the chronic phase of RILD, providing a more precise representation of the progression of RILD. (B) NMR analysis heatmaps displaying metabolite levels at various time points after irradiation and those before irradiation (control). Each column represents one sample, and each row represents one distinct metabolite as indicated. The increased and decreased metabolites are given in red and blue, respectively. (C) Taurine and hypotaurine metabolism was found to be important in RILD progression, and phenylalanine and tyrosine biosynthesis and metabolism were more critical in the chronic stage.

The pathways associated with the significant metabolites involved were identified using the MetaboAnalyst pathway analysis module. Changes in the levels of intermediates during substance metabolism in the liver tissues of mice were used to infer metabolism. Figure 2C shows the altered associated pathways with an impact greater than 0.2 for mice at 3 and 24 h and 8 and 12 weeks postirradiation. The taurine and hypotaurine metabolism pathway significantly changed at each time point, indicating that taurine could serve as a helpful metabolite biomarker for the effect of radiation on the liver.

### Liver Function Enzymes, Histological Staining, and Cytokines in Chronic RILD

Biochemical indices associated with liver function, i.e., AST, ALT, and TBIL levels, were evaluated in mice using an automatic biochemical analyzer. Compared with those in the control group, the levels of AST and ALT in mice at 8 weeks and 12 weeks postirradiation were notably greater. These results suggest significant liver inflammation in the chronic stage after receiving 15 Gy radiation. Additionally, the TBIL levels were higher in mice at 8 weeks and 12 weeks postirradiation than in mice in the control group, indicating the likelihood of liver injury in the chronic stage (Figure 3), which was further supported by histological and molecular markers of progressive hepatic dysfunction.

**Figure 3:**
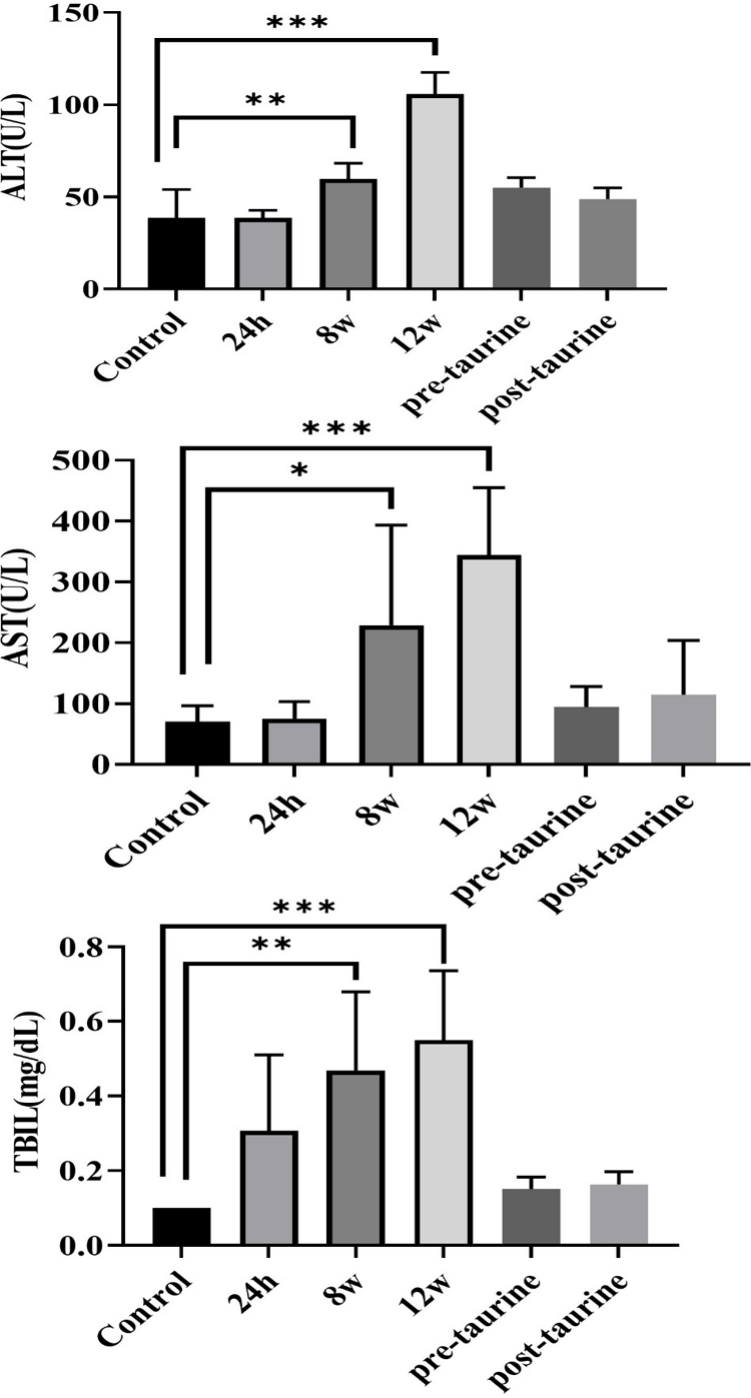
Biochemical enzyme levels associated with liver function at various time points after irradiation and after taurine treatment compared with those at baseline (control). The levels of AST, ALT and TBIL in the groups at 8 and 12 weeks postirradiation were significantly greater than those at baseline (control). Increased AST, ALT, and TBIL levels indicate liver inflammation or liver disease. Compared with those in the control group, the levels of AST, ALT and TBIL were significantly lower in the taurine-treated group than in the taurine-untreated group and not comparable to those in the control group (*, *p* < 0.05; **, *p* < 0.01; ***, *p* < 0.001).

To identify morphological changes and fibrosis in the liver of the mice, H&E and MT staining were conducted on the right lobe of the liver. Mice in the control group exhibited a normal hepatic lobule structure with neatly arranged hepatic cords and normal hepatocytes. However, following radiation exposure, no apparent morphological changes were observed in hepatocytes at 3 and 24 h postirradiation (data not shown). By contrast, at 8 and 12 weeks postirradiation, hepatocytes exhibited enlarged and loosened cytoplasm, as observed in H&E-stained sections. Moreover, MT staining demonstrated a marked increase in collagen deposition at 8 weeks postirradiation (1.88 ± 0.58%, *p* < 0.05), which further escalated at 12 weeks postirradiation (4.17 ± 0.62%, *p* < 0.001), compared with the control group (0.17 ± 0.09%). Representative images of H&E and MT staining are shown in Figure 4A.

**Figure 4:**
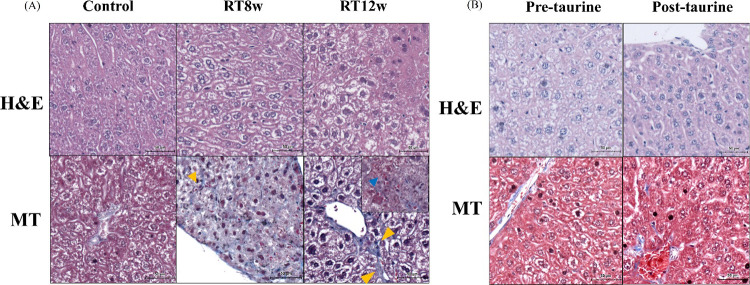
Histopathological changes in the livers of RILD model mice and taurine-treated mice. (A) Hepatic histopathology of postirradiation 8 and 12 weeks was analyzed in mice using H&E and MT staining. Compared with the control, irradiation led to hepatocyte ballooning and degeneration and a loose cytoplasm. MT staining revealed that hepatic fibrosis was strongly exacerbated in mice 12 weeks after irradiation, with sinusoidal obstruction. Yellow arrows indicate collagen, and blue arrows indicate sinusoidal obstruction. (B) In the pretaurine and post-taurine groups, hepatocytes were intact without ballooning. No distinct collagen was produced in the taurine-treated mice. The scale bar represents 50 μm.

The expression of inflammatory and early fibrosis-related cytokines—TNF-α, IL-6, and IL-1β—in the liver parenchyma began to increase at 8 weeks postirradiation, and reached statistically significant levels at 12 weeks, as determined by real-time PCR. In contrast, IL-18 expression remained relatively unchanged throughout the observation period (Figure 5). These findings indicate that a single 15 Gy radiation exposure induced chronic inflammation and contributed to the progression of early stage fibrosis in liver tissues.

**Figure 5:**
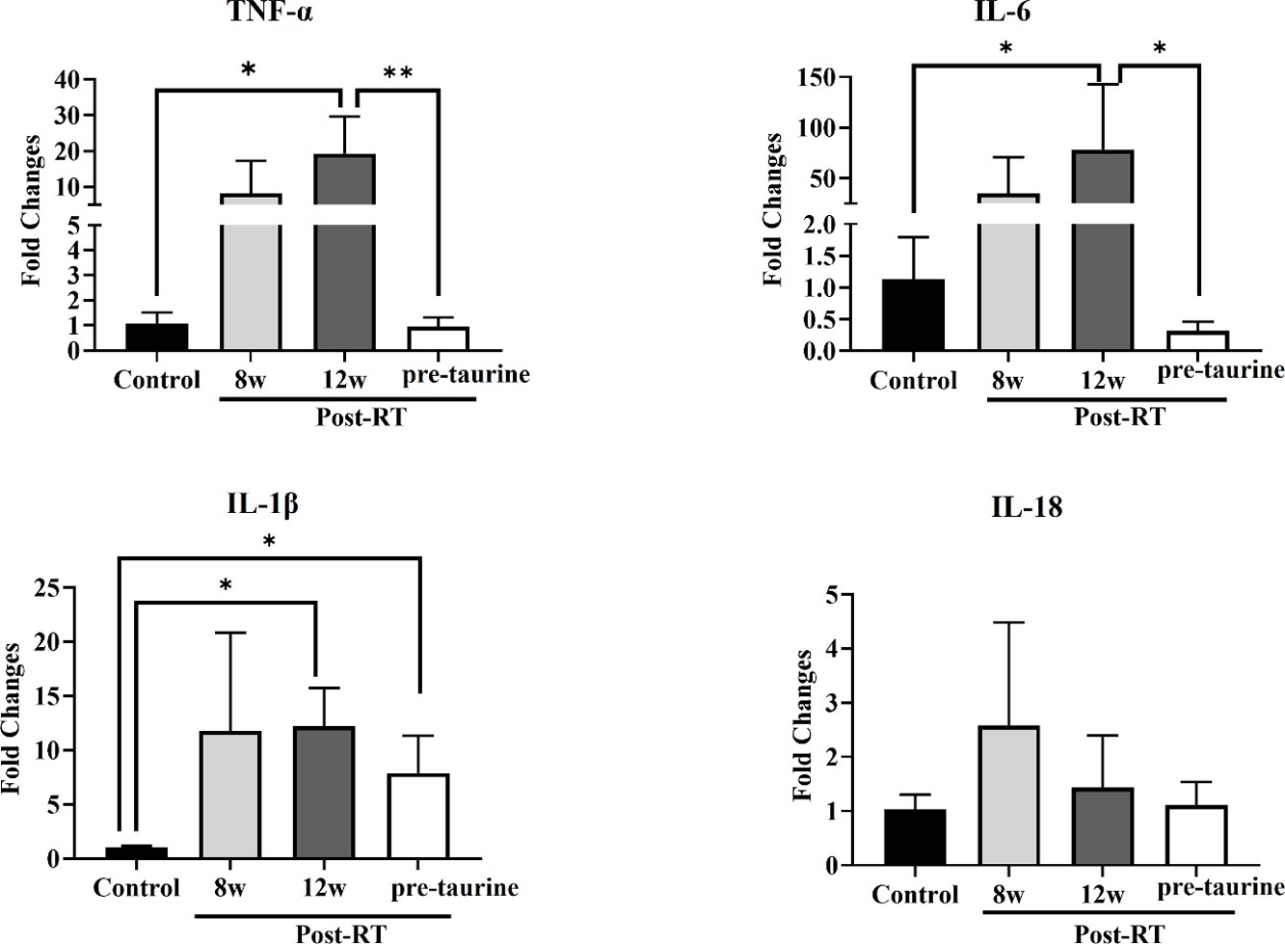
Quantitative results for inflammatory and fibrosis-related cytokines. The levels of TNF-α, IL-6, and IL-1β were significantly elevated at 12 weeks postirradiation compared with those before irradiation. With taurine treatment, the expression of TNF-α and IL-6 decreased and was not comparable to that of the control; the expression of IL-1β was higher than that of the control (*, *p* < 0.05; **, *p* < 0.01).

Taken together, the progression of tissue damage and fibrosis was accompanied by consistent changes in circulating biomarkers. The elevations in AST, ALT, and TBIL reflected the histopathological and molecular evidence of chronic inflammation and early stage fibrosis in the liver. These correlated findings confirm the successful establishment of a chronic RILD mouse model.

### The Impact of Taurine Supplementation on Irradiated Mice

The ^1^H NMR metabolomic results (Table 1) revealed a significant decrease in taurine levels at 8 and 12 weeks postirradiation. To investigate the potential protective effects of taurine supplementation against RILD, we conducted a pilot study in which 2% taurine was administered to mice through drinking water before (pretaurine) and after (post-taurine) irradiation. An analysis of liver enzymes demonstrated that ALT, AST, and TBIL levels were significantly lower in both taurine-treated groups than 12 weeks postirradiation group, with no significant differences observed between these groups and the control group (Figure 3). Histological examination revealed that liver tissues from both taurine-treated groups exhibited more intact morphology and reduced collagen expression (pretaurine: 1.65 ± 0.24, *p* < 0.05; post-taurine: 1.98 ± 0.94, *p* < 0.05) than irradiated mice did at 12 weeks postirradiation only (Figure 4B). However, the degree of collagen in both taurine-treated groups remained slightly greater than that in the control group. The levels of inflammatory cytokines and fibrosis-associated cytokines were assessed before taurine treatment. Compared with the taurine untreated groups, the pretaurine treatment group presented significant reductions in TNF-α and IL-6 levels at 12 weeks postirradiation. No significant differences in TNF-α or IL-6 levels were observed between the control and pretaurine treatment groups; however, a significant difference in the IL-1β level was noted, as shown in Figure 5. To investigate the impact of taurine on liver metabolic changes in RILD, metabolite changes in the pretaurine and post-taurine groups were evaluated with ^1^H NMR and compared with those in the control group. Compared with those in the control group, the levels of taurine in both groups did not significantly differ. Moreover, the levels of phenylalanine and tyrosine in the taurine-treated group were not significantly different from those in the control group, indicating that taurine supplementation effectively reduced the levels of fibrotic metabolic biomarkers. The detailed metabolite changes are listed in Table 2. However, the high concentration of methionine in the taurine treatment groups should be considered (pretaurine, FC = 3.07 and post-taurine, FC = 2.78). Taken together, these findings suggest that taurine supplementation can potentially alleviate RILD.

**Table 2:** Changes in Metabolites after Pre-Taurine Treatment and Post-Taurine Treatment Compared with Baseline (Control)^a^

metabolite	fold change (pretaurine/ctrl)	VIP > 1.1	Wilcox-rank test (p-value)	FDR (q-value)
methionine	3.0715	1.9961	0.015873*	0.19048
mannose	0.39181	1.8958	0.007937**	0.19048
creatinine	0.34563	1.8914	0.095238	0.28571
betaine	3.5426	1.5768	0.095238	0.28571
trimethylamine N-oxide	1.538	1.247	0.22222	0.38095
phenylalanine	0.60826	1.2269	0.095238	0.28571
	fold change (post-taurine/ctrl)			
betaine	3.718	2.1735	0.007937**	0.063492
methionine	2.7811	1.8995	0.007937**	0.063492
creatinine	0.31982	1.8068	0.007937**	0.063492
mannose	0.5806	1.2202	0.095238	0.22857
creatine	1.5954	1.189	0.015873*	0.095238

a *, *p* and *q* < 0.05. **, *p* and *q* < 0.01.

## Discussion

This study investigated the dynamic metabolic changes in irradiated liver tissues using a ^1^H NMR metabolomics platform and revealed the underlying mechanism related to RILD progression. Our findings highlight the changes in taurine levels during acute and chronic stages, suggesting its involvement in the RILD process. Further, morphologic changes in liver tissues were observed as early as 3 h postirradiation and persisted for up to 12 weeks, indicating the progressive nature of radiation-induced liver injury. In vitro results showed that radiation increased ROS production in hepatocyte cells and reduced cell survival (Supplemental Data 2). On the basis of the in vitro results and physiological in vivo data, the potential pathway associated with the metabolic mechanism underlying RILD progression is schematically proposed in Figure 6.

**Figure 6:**
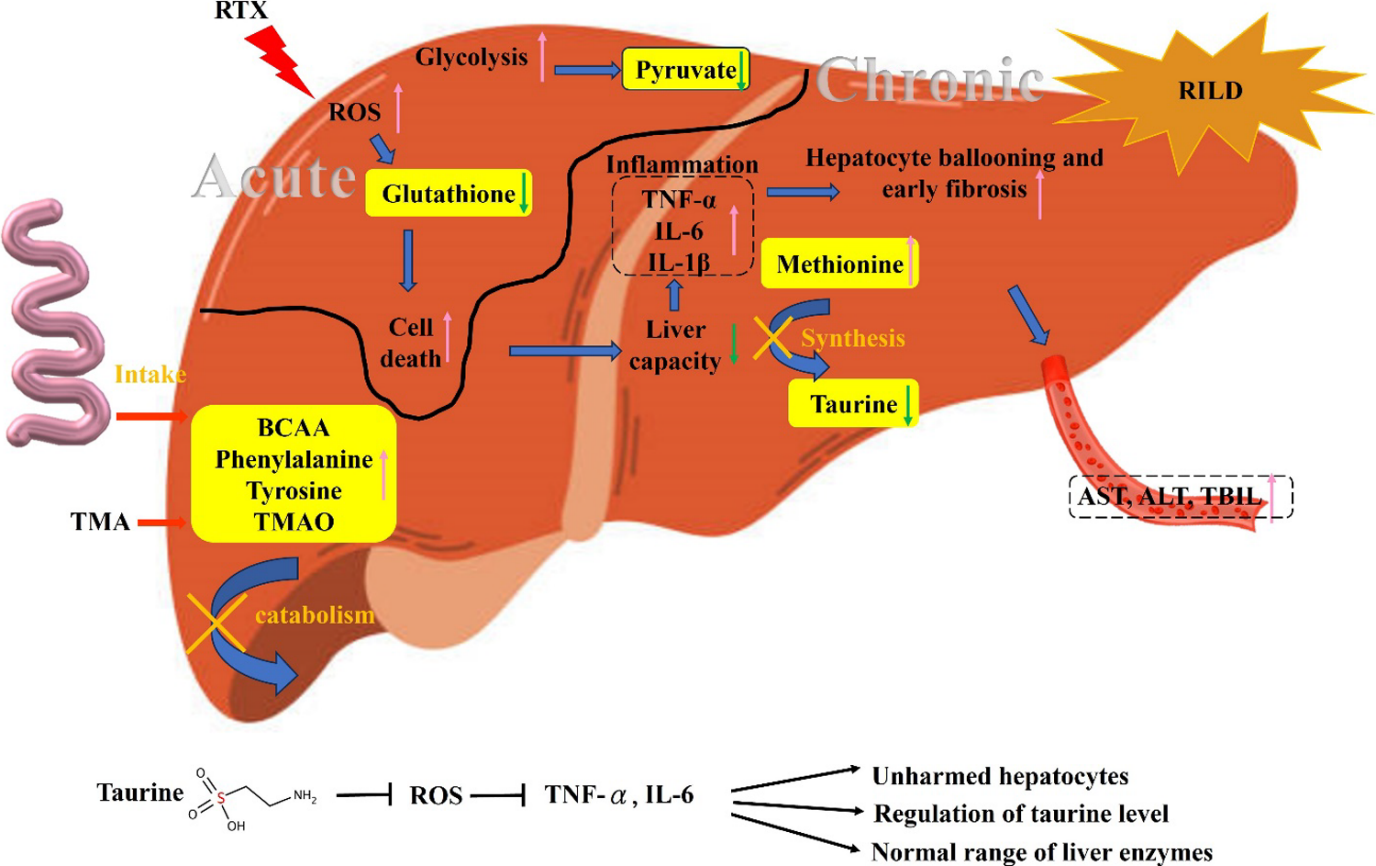
Proposed metabolite changes are associated with RILD progression and the effect of taurine on liver rescue. Radiation induces hepatocytic injury in the acute stage and causes low liver capacity and function in the chronic stage, associated with the dysregulation of multiple metabolic pathways. The level of taurine decreased in the chronic stage due to disrupted methionine metabolism, which decreased the protection of hepatocytes via antioxidants.

Many metabolite levels are altered at different time points and are associated with specific metabolic pathways. In the acute disease stage, metabolite changes were associated with glycolysis and the TCA cycle, taurine and hypotaurine metabolism, and ROS production. In our previous study, gluconeogenesis and glycolysis in the hepatic parenchyma were altered by irradiation in the acute stage, as demonstrated in a murine hepatocellular carcinoma model,^11^ a finding that is consistent with the perturbation of glycolysis in the acute stage after irradiation in the RILD model. Moreover, the study revealed that glutathione and pyruvate levels decreased in the acute stage, implying that the impact of ROS and glycolytic metabolism were early responses to radiation before tissue morphological alterations were observed in H&E staining. The metabolite changes observed in the chronic stage were associated with taurine and hypotaurine metabolism and phenylalanine-tyrosine-tryptophan biosynthesis. Phenylalanine metabolism is suggested to be associated with inflammatory and fibrogenic factors in liver tissues.^24^ The levels of numerous amino acid metabolites are elevated 12 weeks after irradiation, reflecting the loss of catabolism by damaged hepatocytes.^25^

### The Role of Taurine in Ameliorating RILD

Taurine is a small amino sulfonic acid obtained through the diet or synthesized in the liver using the sulfur-containing amino acids methionine and cysteine. In rodent studies, adult rats synthesize approximately 80% of their total body taurine, with the remainder obtained from dietary sources.^26^ Asma Najibi et al. reported a reduction in cellular and mitochondrial taurine levels in bile duct-ligated rats,^27^ which aligns with our findings that liver stress is associated with decreased taurine levels.

Studies have shown that taurine supplementation is helpful in protecting the liver against various harmful substances, such as cyclosporine-A,^28^ acetaminophen,^29^ and alcohol.^30^ In addition, some studies have shown that taurine can reduce liver damage caused by nutrient- and chemical-induced hepatic steatosis, endoplasmic reticulum stress, inflammation, and injury.^30,31^ While its role in RILD has not been thoroughly investigated, El-Maraghi et al. demonstrated a protective effect of taurine against γ-irradiation-induced hepatocyte apoptosis and inflammation in the acute phase.^21^ The chemical structure of taurine, characterized by the presence of a sulfur atom, enables it to function as an antioxidant within biological systems. As an antioxidant, taurine can scavenge ROS, reduce lipid peroxidation, and stabilize biological membranes.^32^ These properties contribute to the mechanism for mitigating the effects of RILD. In our study, we used ^1^H NMR metabolomics to demonstrate that taurine significantly impacted the acute and chronic stages of RILD. We conducted pilot studies to prove that dietary taurine supplementation can reduce the level of RILD in a murine model. Taurine supplementation significantly reduced serum AST and ALT levels and restored the hepatic metabolic profile to a state comparable with nonirradiated controls, suggesting a protective effect during the chronic stage of RILD. When considered alongside the findings of El-Maraghi et al.,^21^ who demonstrated the antiapoptosis and anti-inflammatory role of taurine in the acute phase of irradiation-induced liver injury, our results support the hypothesis that taurine may have protective potential across both the early inflammatory and late fibrotic phases of RILD. While taurine showed protective potential in RILD, its specificity as a biomarker is limited, given its involvement in various liver injuries^33,34^ To clarify its role, future studies will incorporate disease-relevant models, such as CCl_4_-induced liver injury combined with radiation. Additionally, we observed changes in multiple metabolic pathways, including glycolysis, oxidative stress (glutathione), and aromatic amino acids that support a broader metabolic response beyond taurine alone. Figure 6 schematically highlights the critical role of taurine in the progression of RILD. Our findings suggest that taurine supplementation has significant potential as a preventative strategy for patients with liver and upper abdominal malignancies receiving radiation therapy. We believe that by linking metabolite profiling with functional outcomes, our study provides new insight into the metabolic mechanisms underlying RILD progression and identifies taurine as a potential target for intervention to mitigate chronic radiation-induced liver injury.

### Phenylalanine and Tyrosine in the Chronic Stage of RILD

The ^1^H NMR metabolomic results revealed significantly increased levels of phenylalanine and tyrosine during the chronic stage of RILD. Moreover, hepatic collagen staining revealed hepatic fibrosis in the chronic stage. Some studies have suggested that phenylalanine and tyrosine intake through the diet can aggravate hepatic fibrosis.^35^ In addition, in our study, with taurine treatment, decreased phenylalanine and tyrosine levels in liver tissues corresponded to the normal status of liver enzymes, tissue morphology, and cytokine levels. Hepatic fibrosis is caused by HSCs that secrete fibrogenic factors. These factors promote collagen production by portal fibrocytes, fibroblasts, and bone marrow-derived myofibroblasts, leading to the initiation, progression, and regression of liver fibrosis.^36^ However, additional studies are needed to understand the relationships between hepatic fibrosis-associated amino acids, i.e., phenylalanine and tyrosine, and fibrogenic factors from HSCs.

### Limitations and Clinical Application

One limitation of this study is that the murine RILD model, induced by a single irradiation dose of 15 Gy, did not fully develop severe fibrosis or cirrhosis within the 12-week observation period. While the acute stage of RILD was observed and is consistent with our previous findings,^11^ the chronic stage, which involves a greater risk of severe liver damage, has been shown to require irradiation doses exceeding 30 Gy over 6–20 weeks.^13,37^ As a result, collagen accumulation and the fibrosis-associated cytokine IL-18 did not significantly increase in this model. Future studies should consider employing 30 Gy irradiation within a 12-week period to better investigate severe RILD.

Blood, urine, feces, and tissues are often analyzed in metabolomic studies;^38^ however, blood collection is more feasible for patient studies. This study performed metabolomic analysis using liver tissues, which may limit its applicability in clinical settings. However, positive correlations between plasma and liver tissue metabolites have been reported.^39,40^ Therefore, our findings hold significant potential for translation into clinical trials. In addition, the establishment of a platform for circulating biomarker detection will facilitate clinical applications extensively in the future. More importantly, this study could help guide the use of taurine supplementation to diminish or prevent the risk of classical RILD in liver cancer patients undergoing radiation therapy. However, in our study, only a single-fraction SBRT was applied to mice with healthy livers,^5,41^ and variations in irradiated liver volume were not assessed. Therefore, caution is warranted when extrapolating our findings to different clinical fractionation strategies and varying liver volumes. Interestingly, our healthy mouse model exhibited both classical and nonclassical features, potentially due to species differences or the absence of pre-existing liver disease. Although the clinical classification of RILD is well described, metabolic distinctions between classical and nonclassical RILD have not been clearly defined in the literature. Our findings may offer preliminary insights into this underexplored area. Future studies incorporating disease-relevant models, such as liver fibrosis or hepatitis, may enhance our understanding of the pathogenesis and spectrum of nonclassical RILD following SBRT.

## Conclusions

This study identified pivotal metabolites associated with the effects of ROS, glycolysis, inflammation, and early fibrosis caused by radiation and the mechanisms associated with RILD. The findings demonstrated that taurine is a potential target for alleviating fibrosis in irradiated liver tissues.

## Supplementary Material



## Data Availability

The NMR data have been deposited in Metabolomics Workbench (www.metabolomicsworkbench.org) repositories, under 4773/DataTrackID5905.

## Notes

Irradiation field of view (FOV, 10 × 10 mm), indicated by the green box, partially overlapped with the liver parenchyma, radiation-induced ROS production and reduced viability in AML12 hepatocytes (PDF)

## Abbreviations

(AST): Aspartate aminotransferase
(ALT): Alanine aminotransferase
(BCAAs): Branch-Chain Amino Acids
(H&E): Hematoxylin-eosin
(HSC): Hepatic stellate cells
(MT): Masson’s trichrome
(NMR): Nuclear magnetic resonance
(PLS-DA): Partial least-squares discriminant analysis
(RILD): Radiation-induced liver disease
(ROS): Reactive oxygen species
(TBIL): Total bilirubin
(TCA): Tricarboxylic acid
(TSP): Trimethylsilylpropanoic acid
(VIP): Variable importance in projection
