# Supplementary material for: Taurine as a Protective Metabolite in Radiation-Induced Liver Disease: Evidence from 1H NMR Metabolomics
Source: J Proteome Res. 2025 Aug 11;24(9):4708–18. doi: 10.1021/acs.jproteome.5c00398 (PMC12418501; doi:10.1021/acs.jproteome.5c00398)

Taurine as a Protective Metabolite in Radiation-Induced Liver Disease: Evidence from ^{1}H NMR Metabolomics

Yi-Hsiu Chung[1], Chi-Chang Weng[2], Fujie Jhang[1], Gigin Lin[2,3,4], Ching-Fang Yu[2,5,6], and Fang-Hsin Chen[7]*

** Ching-Fang Yu is the co-corresponding author*

1.Department of Medical Research and Development, Research Division, Chang Gung Memorial Hospital at Linkou, Taoyuan, 33382, Taiwan

2.Department of Medical Imaging and Radiological Sciences, Chang Gung University, Taoyuan, 33302, Taiwan,

3.Department of Medical Imaging and Intervention, Chang Gung Memorial Hospital at Linkou, Taoyuan,33382, Taiwan

4.Clinical Metabolomics Core and Imaging Core Laboratory, Institute for Radiological Research, Chang Gung Memorial Hospital at Linkou and Chang Gung University, Taoyuan,33382, Taiwan

5.Research Center for Radiation Medicine, Chang Gung University, Taoyuan 33302, Taiwan

6.Department of Radiation Oncology, Chang Gung Memorial Hospital Linkou Branch, Taoyuan 33382, Taiwan.

7.Institute of Nuclear Engineering and Science, National Tsing Hua University, Hsinchu, 300044, Taiwan

Table of contents:

Supplemental Data 1. The irradiation field of view (FOV, 10 × 10 mm), indicated by the green box, partially overlapped with the liver parenchyma.

Supplemental Data 2. Radiation-induced ROS production and reduced viability in AML12 hepatocytes.

Supplemental Data 1. The irradiation field of view (FOV, 10 × 10 mm), indicated by the green box, partially overlapped with the liver parenchyma.

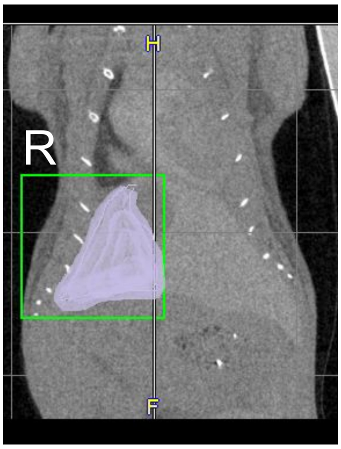

Supplemental Data 2. Radiation-induced ROS production and reduced viability in AML12 hepatocytes. (A) ROS levels were significantly increased 15 minutes after 3 Gy irradiation ($p < 0.05$, $n = 3$). (B) Cell viability significantly decreased at 48 and 72 hours postirradiation ($p < 0.05$, $n = 3$), indicating radiation-induced oxidative stress and reduced hepatocyte survival.

(A)

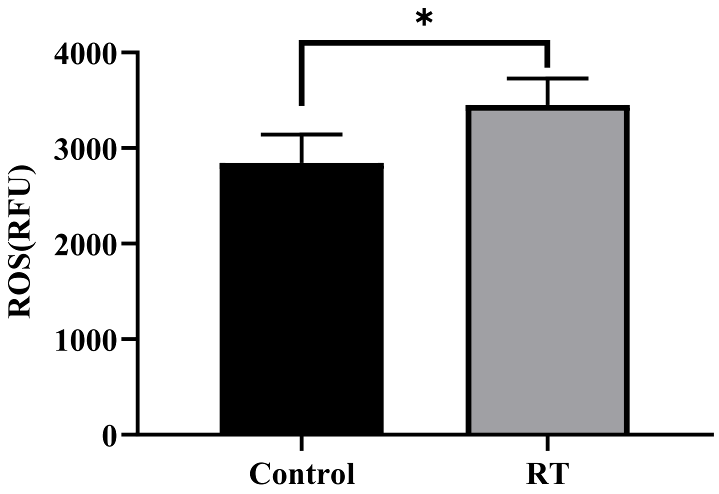

(B)

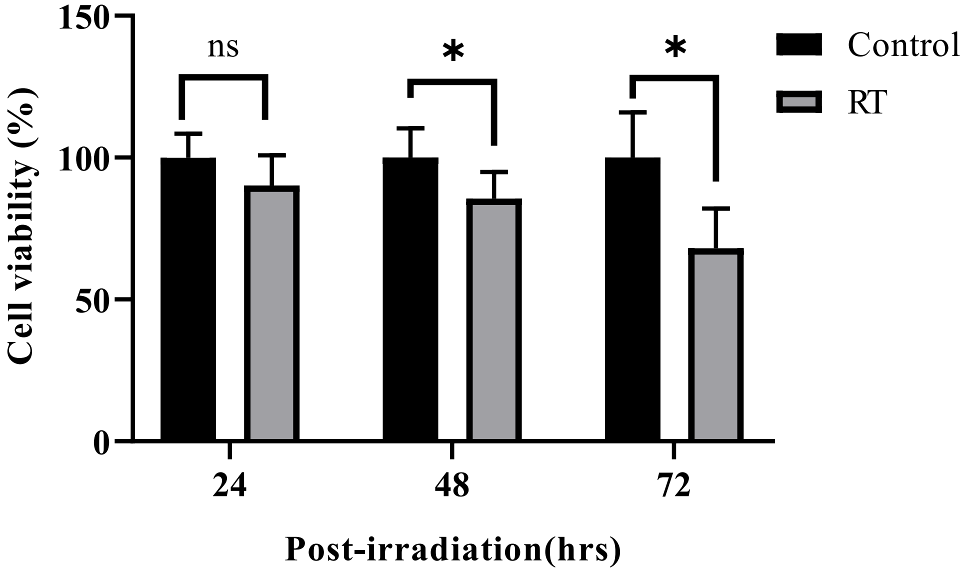

Supplement: Supplementary file 1 [file pr5c00398_si_001.pdf]
